# Programming temporal shapeshifting

**DOI:** 10.1038/ncomms12919

**Published:** 2016-09-27

**Authors:** Xiaobo Hu, Jing Zhou, Mohammad Vatankhah-Varnosfaderani, William F. M. Daniel, Qiaoxi Li, Aleksandr P. Zhushma, Andrey V. Dobrynin, Sergei S. Sheiko

**Affiliations:** 1 Department of Chemistry, University of North Carolina at Chapel Hill, Chapel Hill, North Carolina 27599-3290, USA; 2 Department of Polymer Science, University of Akron, Akron, Ohio 44325-3909, USA

## Abstract

Shapeshifting enables a wide range of engineering and biomedical applications, but until now transformations have required external triggers. This prerequisite limits viability in closed or inert systems and puts forward the challenge of developing materials with intrinsically encoded shape evolution. Herein we demonstrate programmable shape-memory materials that perform a sequence of encoded actuations under constant environment conditions without using an external trigger. We employ dual network hydrogels: in the first network, covalent crosslinks are introduced for elastic energy storage, and in the second one, temporary hydrogen-bonds regulate the energy release rate. Through strain-induced and time-dependent reorganization of the reversible hydrogen-bonds, this dual network allows for encoding both the rate and pathway of shape transformations on timescales from seconds to hours. This generic mechanism for programming trigger-free shapeshifting opens new ways to design autonomous actuators, drug-release systems and active implants.

## Abstract (web summary)

Actuation of shape-shifting materials has typically required an external trigger. Here, the authors design a shape-memory hydrogel, regulated by a dual network of covalent and temporary hydrogen bonds, whose actuations are encoded by an intrinsic temporal mechanism.

## Graphical abstract

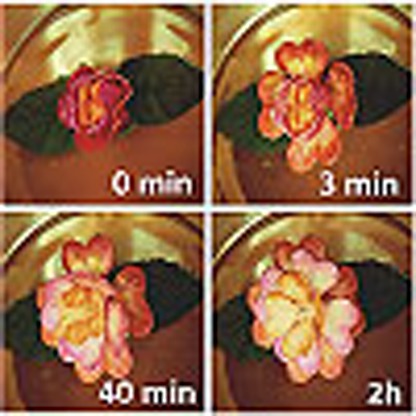

Shape is integral to matter, impacting colour, motility, strength and packing^1^,^2^,^3^,^4^,^5^,^6^,^7^. In recent years, various shape-memory and shape-changing materials have been developed, enabling programmable actuations in response to external triggers such as a distinct alteration of temperature, pH, light, chemicals and sound^8^,^9^,^10^,^11^,^12^,^13^,^14^,^15^,^16^,^17^. These materials exhibit both one-way shifting between multiple shapes and two-way reversible transformations^18^,^19^,^20^,^21^,^22^, but these changes happen only in response to external trigger events. This requirement prompts a challenging question: Can we design a material that, like a mechanical watch (Fig. 1a), possesses a built-in energy-storage device (spring) and gear-analogues to regulate the rate of energy release? If so, we can envision biomedical implants that undergo an autonomous sequence of shape changes with pre-determined rates.

Here, to realize the watch analogy, in line with the recent studies of temporal control of snapping transformations in bistable structures^23^, we consider a dynamic polymer network comprising two types of crosslinks (Fig. 1a): (i) a low concentration of covalent crosslinks to form a network, which when deformed, stores the elastic energy that drives shape recovery; (ii) a high concentration of reversible physical crosslinks, which temporarily lock-in new shapes caused by deformations. Due to the time-dependent reorganization of the reversible physical crosslinks, the temporal programming of this dual network enables both sequential and reversible shape transformations, which occur with pre-determined rate and without applying an external trigger.

## Results

### Theoretical analysis of reversible network

**Figure 1:**
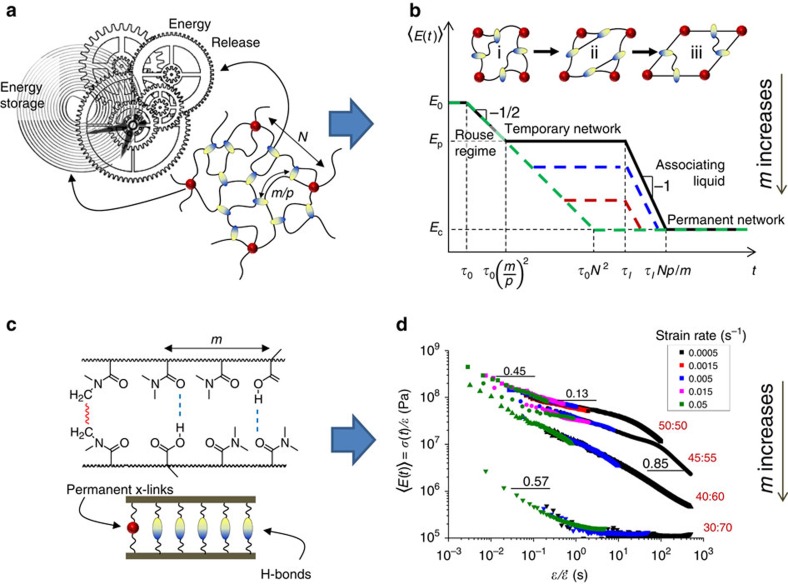
Dynamic networks. (**a**) Mechanical watch machinery—an inspiration for the design of a dual polymer network with chemical crosslinks (dots) for energy storage and reversible physical crosslinks (ovals) for controlled energy release: *N* and *m*/*p*—number of monomeric units between the chemical and physical crosslinks, where *m*—degree of polymerization between associating groups and *p*—degree of conversion of the associating groups into crosslinks. (**b**) At constant strain rate 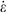, the evolution of the time-average network Young's modulus 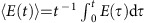 during deformation 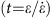 is controlled by two characteristic relaxation times: *τ*_0_—monomeric timescale, *τ*_l_—lifetime of physical crosslinks. At (*m*/*p*)^2^*τ*_0_<*t*<*τ*_l_, Rouse-like relaxation of the network strands 
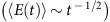
 is followed by the physical network modulus plateau at *E*_p_≅*E*_0_*p*/*m*. After pausing at the plateau (ii), the deformation process continues through recurring dissociation/re-association of the physical crosslinks. In this time window (*τ*_l_<*t*<(*Np*/*m*)*τ*_l_∼hours), the system behaves as a viscous liquid 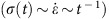, while the physical network rearranges in a new stress-free configuration (iii)^25^. At *t*>(*Np*/*m*)*τ*_l_, the ‘flow' is terminated by the covalent network resulting in the second modulus plateau at *E*_c_≅*E*_0_/*N*. (**c**) Methacrylic acid (MAAc)-*co*-*N,N*-dimethylacrylamide (DMAA) hydrogels include covalent crosslinks (dot) and hydrogen-bonds between MAAc and DMAA groups (ovals), where *m* depends on the copolymerization ratio. (**d**) 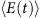
was extracted from stress-strain relations 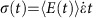, measured at 3 °C at constant 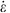, ranging from 0.0005 to 0.05 s^−1^ (Supplementary Fig. 3). The standard deviation of the average of three separate experiments at each strain rate is within 5%. The four curves correspond to different MAAc:DMAA molar ratios (50:50, 45:55, 40:60 and 30:70), which determine the physical crosslinking density. The slopes of the curve at different time windows are indicated above the horizontal short lines. Consistent with the theoretical prediction, *E*_p_ decreases with increasing *m*. The 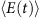 analysis was chosen because time–temperature superposition fails to describe dynamics of the associating networks (Supplementary Fig. 4) governed by two distinct relaxation processes^24^: segmental motions within polymer chains (*τ*_0_) and dissociation of H-bonds (*τ*_l_). Validity of the linear viscoelasticity approximation has been verified (Supplementary Equation 29; Supplementary Fig. 5).

To delineate contributions from the chemical and physical networks to shape control, Fig. 1b presents a theoretical analysis of stress evolution versus time in a dual network undergoing deformation at constant strain rate (Supplementary Discussion, Supplementary Equations 1–28, Supplementary Figs 1 and 2). During deformation (shape programming), the physical network breaks and reforms in a new stress-free configuration^25^, which results in two plateaus at the Young's moduli of the physical (*E*_P_) and chemical (*E*_c_) networks, respectively. During shape recovery, the rearranged physical network counteracts the restoring force from the chemical network (*E*_p_ versus *E*_c_) and secures the temporary shape. The lifetime of the physical crosslinks should be longer than the relaxation time of the chemical network strands to ensure shape recovery over relevant timescales (seconds to hours). The reversibility of physical crosslinks ensures steady shapeshifting and full restoration of the sample's original shape and mechanical properties.

### Design of dual network hydrogel

To prove the concept, we have synthesized hydrogels by copolymerization of *N,N*-dimethylacrylamide (DMAA) and methacrylic acid (MAAc), which yields a dense H-bonded network integrated with a loose chemical network (Fig. 1c). Due to the hydrophobic methyl substituents, the MAAc and DMAA groups undergo strong association^26^,^27^, which leads to the formation of H-bonded clusters yielding particularly tough hydrogels with mechanical properties on par with living cartilage^28^. For example, a 50:50 MAAc-*co*-DMAA sample (70 wt% water, pH=3, *T*=22 °C) gives a *E*∼30 MPa, fracture energy of ∼9,300 J m^−2^, and extensibility of ∼600%. These polymer gels undergo full recovery of both shape and all mechanical properties at controlled rates with no external trigger, even after ∼600% pre-rupture deformation (Supplementary Fig. 6).

In agreement with the theoretical predictions (Fig. 1b), mechanical properties of these gels depend on the crosslinking density of the physical network. In Fig. 1d, 50:50 MAAc:DMAA (densest physical network) displays two relaxation transitions separated by a plateau: (i) Rouse relaxation of the network strands (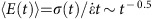) at *t*∼ms (*τ*_0_<*t*<(*m*/*p*)^2^*τ*_0_) and (ii) yielding of the physical network due to dissociation of the H-bonds 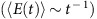 at *t*∼min. From the measured moduli of the physical and chemical networks (Supplementary Fig. 7), we have extracted *N*≅120 and *p*/*m*=*E*_p_/*NE*_c_≈1, respectively. Within limits of scaling approximation, these molecular parameters predict characteristic timescales *t*=*τ*_1_≈10 s and 
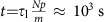
 (Fig. 1b), which are of the same order of magnitude as observed in Fig. 1d. Upon decreasing fraction of the associating groups, the plateau modulus decreases (samples 45:55 and 40:60) and eventually merges with *E*_c_≅10^5^ Pa of the permanent network (30:70). Unlike the theoretical prediction in Fig. 1b, the temporary network plateau is not distinct due to the crossover of the Rouse and Associating Liquid regimes. We could potentially extend the onset of the liquid regime by strengthening the lifetime of H-bonds, but this would shift the shape recovery process to prohibitively long times. In a separate set of experiments, we have shown that *E*_c_ increases with chemical crosslink density (Supplementary Fig. 8) and *E*_p_ decreases with temperature (Supplementary Fig. 7) due to the dissociation of H-bonds monitored by IR absorption (Supplementary Fig. 9). Temperature affects both concentration and strength of H-bonds due to the entropic contribution to the bond free energy.

### Controlling the rate and pathway of shapeshifting

**Figure 2:**
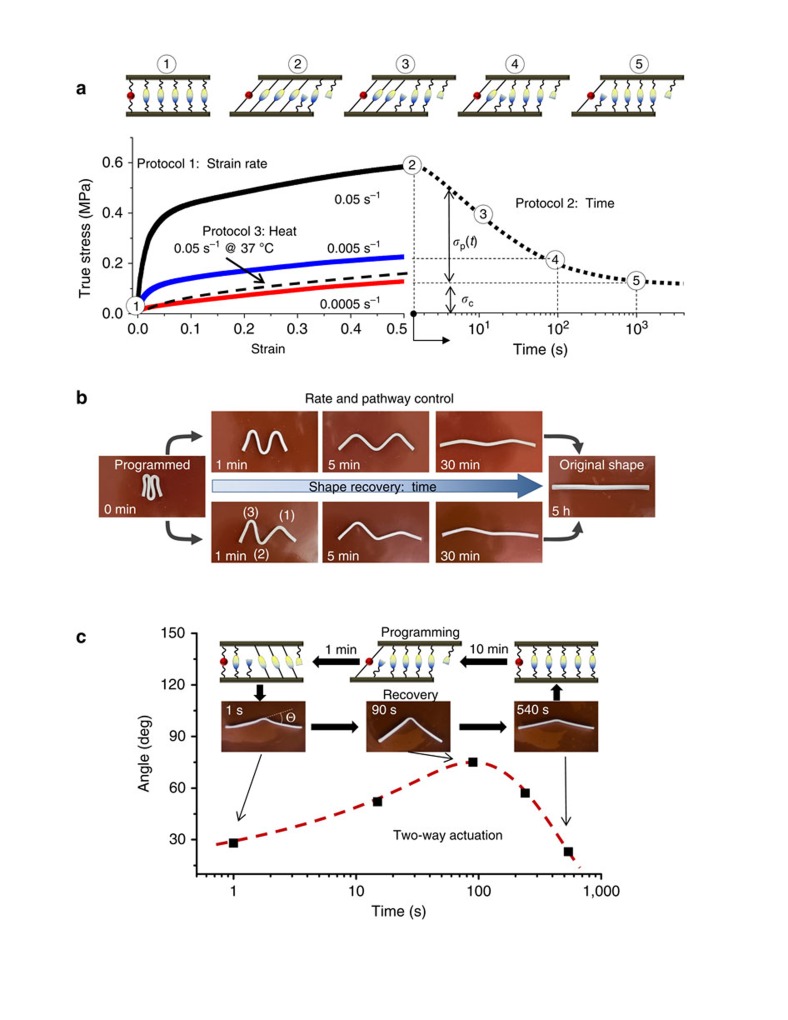
Different programming protocols and shape-recovery pathways. (**a**) Three different shape programming protocols produce identical deformation states of a dual network (uniaxial extension, *ɛ*=0.5): (i) Deformation at different strain rates as indicated (solid lines, *T*=25 °C), (ii) Stress relaxation for controlled times (dotted line, *ɛ*=0.5, 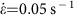, 25 °C), and (iii) Deformation at a higher temperature (dashed line, 37 °C). Top panel: network re-arrangement at different stages of stress relaxation (–). The simple shear schematic is a conventional way for picturing deformation under constant volume conditions. The solid and dashed ovals correspond to the original and re-associated H-bonds, respectively. During shape recovery, the strained chemical network drives reverse dissociation/association of these transient H-bonds towards the original shape. (**b**) A triple hairpin demonstrates the ability to control both rate and pathway of shape recovery by adjusting the programming time (*t*_p_) of the individual angles. Upper panel: all three angles were programmed identically (Protocol 2: *t*_p_=100 min) and therefore, unfold simultaneously. Lower panel: The angles were held for different times ((1):1 min, (2):10 min, (3):100 min), which resulted in sequential unfolding with shorter *t*_p_ resulting in faster recovery. Both programming and recovery were conducted under the same conditions (oil, *T*=22 °C). (**c**) Reversible shapeshifting of a hairpin was programmed in two steps: (i) folding and stress relaxation for 10 min, followed by (ii) unfolding and stress relaxation for 1 min. After release of the external force, the hairpin undergoes shape recovery through folding-unfolding motions (Supplementary Movie 1) under constant surrounding conditions (*T*=22 °C). All samples are 50:50 MAAc-*co*-DMAA gel (70 wt% water). To control water evaporation, all deformation and shape-recovery tests were conducted in either silicone oil or pH 3 water (Supplementary Fig. 10).

Unlike conventional stimulation methods of shape recovery, for example, by increasing temperature above a specific trigger point, below we demonstrated a new capability to vary the shapeshifting rate by changing programming protocols without a specific alteration of the environmental conditions. Reversible networks provide a range of different protocols for encoding both rate and pathway of shapeshifting (Fig. 2a). Protocol 1 (deformation at controlled strain rates) and Protocol 2 (stress relaxation at controlled times) yield identically stressed samples (see horizontal dashed lines) for the same total programming time (*t*_p_), including the durations of the deformation 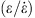 and stress relaxation processes. Protocol 3 (deformation at higher temperature) is a short cut with respect to Protocols 1 and 2, as it allows for faster re-arrangement of the strained physical network, while yielding a shape with the same ‘shape-memory clock'. Although the molecular arrangement of differently programmed networks may be different, the identical true stress at the same strain values suggests the same average crosslink density and leads to similar kinetics of shape recovery (Supplementary Fig. 11a). The restoring stress *σ*(*t*, *ɛ*) is a sum of *σ*_c_(*ɛ*)—strain-controlled stress from the permanent chemical network and *σ*_p_(*t*)—time-dependent stress of the temporary physical network (Fig. 2a). Samples that are allowed to relax for a longer time have lower internal stress due to re-arrangement of the H-bonded network, and therefore, display slower shape recovery. This effect is demonstrated in Fig. 2b, where a triple hairpin unfolds along two different pathways depending on programming time applied to each individual angle. Note that the observed shape transformations are not triggered by heating samples above a certain threshold temperature, as no thermal transitions are observed in this temperature range (Supplementary Fig. 12). The shape recovery occurs spontaneously at temperatures, which can be equal to, higher, and even lower than the programming temperature. However, the recovery temperature has an effect on the rate of shape recovery (Supplementary Fig. 11b). Unlike the conventional shape-memory polymers which can display partial shape recovery below their programming temperature, our systems restore 100% of the original shape after large deformation (up to 600%) even at *T*<*T*_programming_. Furthermore, due to the temporary stress storage in the transient network, these polymeric gels can be programmed for sequential shapeshifting in reverse directions (Fig. 2c; Supplementary Movie 1). Effectively, the applied programming process results in a system with two transient sub-networks strained in opposite directions. This triple shape-memory (one-way reversible shapeshifting) behaviour occurs at constant temperature, that is, without using a sequence of external triggers as is used in conventional triple shape transitions^21^. Since the programming and recovery process occurred under the same environmental conditions, we observe a virtually trigger-free shape-memory behaviour. The presented findings suggest that both shape and viscoelasticity of our gels are determined by reversible reorganization of the relaxation of transient H-bonded networks.

**Figure 3:**
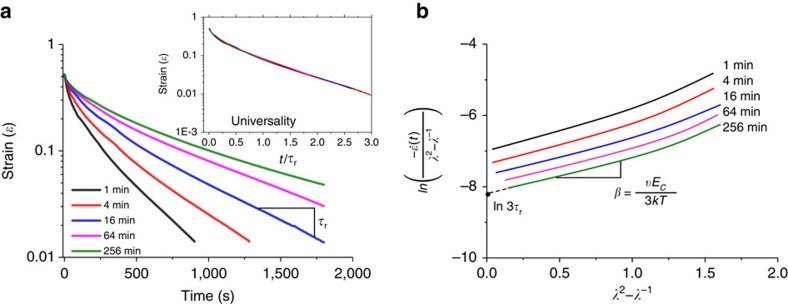
Controlling rate of shapeshifting. (**a**) Kinetics of shape recovery (50:50 MAAc-*co*-DMAA gel, 70 wt% water, Protocol 2: uniaxial extension, 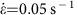, *ɛ*=0.5, *T*=25 °C) for different programming times *t*_p_ as indicated. Inset: The strain recovery curves collapse on a single curve upon time normalization *t*/*τ*_r_. (**b**) Plotting rate of strain recovery versus strain (Supplementary Equation 37) allows extraction of activation volume and lifetime of the physical crosslinks (Supplementary Fig. 14) based on a ‘standard linear solid' model (Supplementary Equations 30–40).

To examine a universal relationship between object properties (shape) and molecular properties (network relaxation), we measured the kinetics of shape recovery (Fig. 3a) and examined its correlations with the modulus variations in Fig. 1d. After a quick initial drop in strain, shape recovery follows a single exponential decay with a characteristic time *τ*_r_, which increases with programming time (Fig. 3a). Upon plotting strain versus *t*/*τ*_r_, all curves collapse onto a single curve (inset in Fig. 3a), which points to universality of the molecular mechanisms responsible for the shapeshifting. A shape-recovery process corresponds to a creep test at zero external force, which can be described with a two-network model (Supplementary Discussion; Supplementary Equations 30–40; Supplementary Fig. 13). By using Eyring's notion of stress-induced shift in bonds lifetime^29^, the model describes the shape-recovery rate using two fitting parameters: *β*=*vE*_c_/(3*kT*) and *τ*_r_ (Supplementary Equation 37). All curves in Fig. 3b show the same slope (*β*=0.9±0.1), whereas the zero-strain intercept (ln(3*τ*_r_)) depends on the programming time. Note that for long programming times, the recovery time levels off at *τ*_r_≈1,200 s (Supplementary Fig. 14). From the obtained fitting parameters and independently measured *E*_c_=250 kPa and *E*_p_/*E*_c_≅130 (Supplementary Fig. 8f), we find an activation volume of *v*=34±4 nm^3^ and lifetime of the physical crosslinks *τ*_1_≅*τ*_r_*E*_c_/*E*_p_=10±2 s. These times agree with the boundaries of the associating liquid regime (Fig. 1b) ranging from *t*=*τ*_1_∼10 s to 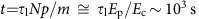 obtained from the modulus evolution 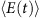 during programming (Fig. 1b,d), and the large activation volume is consistent with the clustering of H-bonds^28^.

### Delayed shape transformation

**Figure 4:**
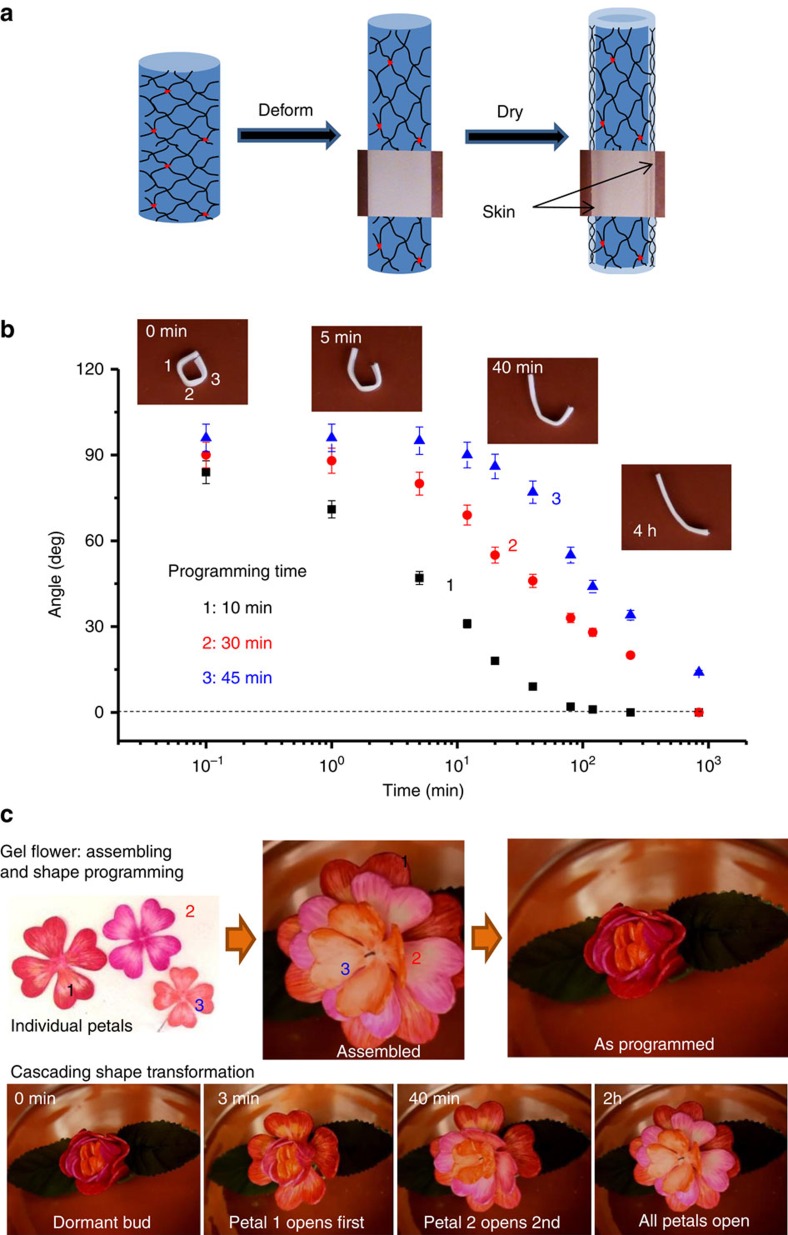
Sequential recovery of dormant shapes. (**a**) Programming protocol: a sample is deformed and held in air to allow partial evaporation of water. An outer skin with low water concentration and high H-bonding strength forms upon drying and fastens the deformed shape. (**b**) Three angles programmed in air for different times (1:10 min; 2:30 min; 3:45 min) undergo spontaneous unfolding with different delay times (pH 3, 22 °C) (Supplementary Movie 2). The error bars indicate the standard deviation for the average of three separate experiments. (**c**) Upper panel: three petal-shape sheets of different sizes are cut from 50:50 MAAc-*co*-DMAA gel (70 wt% water) and coloured for easy distinction. The petals are then assembled and folded in air for different times (1:1 min; 2:10 min; 3:30 min) to create a dormant ‘bud'. Lower panel: sequential ‘blooming' after immersing the programmed ‘bud' in pH 3 buffer at 22 °C (Supplementary Movie 4).

As shown in Fig. 2b,c, control of the recovery rate allows for programming of different shapeshifting pathways. However, for all protocols discussed above, onset of shape recovery occurs instantly after the release of the external force. The lack of an induction period limits applications that require a controllable lag-time for the deployment of a shape-programmed object. Theoretically, this can be resolved by introducing a network with infinite lifetime during programming and then gradually breaking the network during shape recovery. We resolved this issue by simply introducing a thin skin of dehydrated gel on the surface of a programmed sample through partial drying in ambient air (Fig. 4a). The recovery process is halted until the physical network returns to its dynamic state via re-immersion into an aqueous environment. Controlling the skin thickness allows for encoding a well-defined dormant period enabling shape transformation in a distinct step-wise fashion inside stable closed environments (Fig. 4b; Supplementary Movies 2 and 3; Supplementary Fig. 15). Adding temporal control over the onset time of shapeshifting is a vital advancement, which enables creation of cascading actuator assemblies displaying control of both the initiation and rate of multiple actuations. Figure 4c shows sequential ‘blooming' of an artificial flower, which has been assembled from individually programed petals (Supplementary Movie 4).

## Discussion

In conclusion, a dual network hydrogel system has been designed to integrate energy storage and controlled energy release, which enables sequential shape transformations without applying an external trigger. Such systems rely on developing a dual network with dynamic bonds, which satisfy the following requirements: (i) a high concentration to provide rigidity to offset the restoring force of the chemical network and secure a temporary shape, (ii) a suitable lifetime, which should be relatively short-lived to allow for spontaneous reconfiguration of the dual network upon release of an external force, and longer than the Rouse relaxation time of the chemical networks strands to ensure shape recovery over practically relevant timescales (seconds to hours). The outlined molecular mechanism of temporal shapeshifting is generic and can be applied to other types of dynamical networks, once the dynamic bonds meet the above requirements^30^,^31^,^32^. Combined with the recent developments in 3D printing of complex shapes^33^, these time- and route-programmable materials have great potential in biomedical engineering enabling autonomous actuation and minimally invasive surgical procedures^34^,^35^,^36^,^37^,^38^.

## Methods

### Hydrogel preparation

Dual network hydrogels were synthesized by a one-step copolymerization of DMAA and MAAc with different molar ratios, while the total monomer concentration was 33 wt%. A mixture of above monomer aqueous solution with 0.5 mol% ammonium persulfate and *N,N,N'N'*-tetramethylethylenediamine (in a concentration relative to the total monomer concentration) was poured into a glass mold with a PDMS spacer and then polymerized at room temperature for 48 h. To achieve a high crosslink density of the physical network for controlling shape-fixation and -recovery rate, we targeted *m*=1–2.5 through variation of MAAc:DMAAm ratio during polymerization and *p*≅1 by using low pH=3 of the solvent.

### Materials characterization

The mechanical tests were carried out on a Dynamic Mechanical Analysis (RSA-G2, TA Instrument) with an Immersion Clamps. Samples with a thickness of 1.6 mm were cut into dogbone shape (DIN 53504-S3, 2 mm in width with an initial length of 12 mm). To enhance reproducibility, all of the mechanical tests were performed in silicone oil with temperature control. Samples were stretched at a certain strain rate at a defined temperature. For shape programming, sample was stretched to a strain of 50% at a certain strain rate and then held at 50% strain for a defined time. Next, the sample was set to isoforce mode where the external force was set to be constantly at zero and then the strain change over time during the recovery process was recorded.

Further details on the methods are available in the Supplementary Methods.

### Data availability

The data that support the findings of this study are available from the corresponding author on request.

## Additional information

**How to cite this article:** Hu, X. *et al*. Programming temporal shapeshifting. *Nat. Commun.* **7,** 12919 doi: 10.1038/ncomms12919 (2016).

## Supplementary Material

Supplementary Information Supplementary Figures 1-15, Supplementary Discussion, Supplementary Methods and Supplementary References.

Supplementary Movie 1 Two-way shape transformation

Supplementary Movie 2 Sequential shape recovery from 2-D square to 1-D line

Supplementary Movie 3 Step-wise shape recovery from 3-D box to 2-D sheet

Supplementary Movie 4 Blooming "flower" of hydrogel
