# Supplementary material for: Programming temporal shapeshifting
Source: Nat Commun. 2016 Sep 27;7:12919. doi: 10.1038/ncomms12919 (PMC5052638; doi:10.1038/ncomms12919)
Supplement: Supplementary Information — Supplementary Figures 1-15, Supplementary Discussion, Supplementary Methods and Supplementary References. [file ncomms12919-s1.pdf]

Supplementary Figures

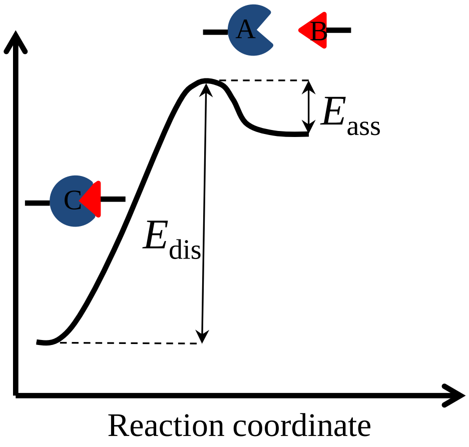

Supplementary Figure 1 | Schematic representation of the associating bond formation.

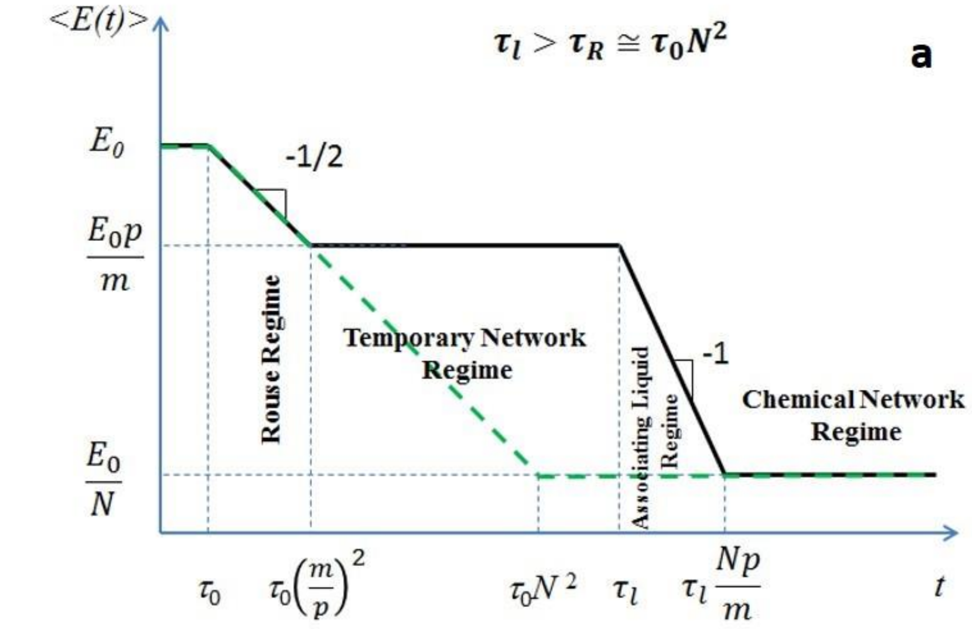

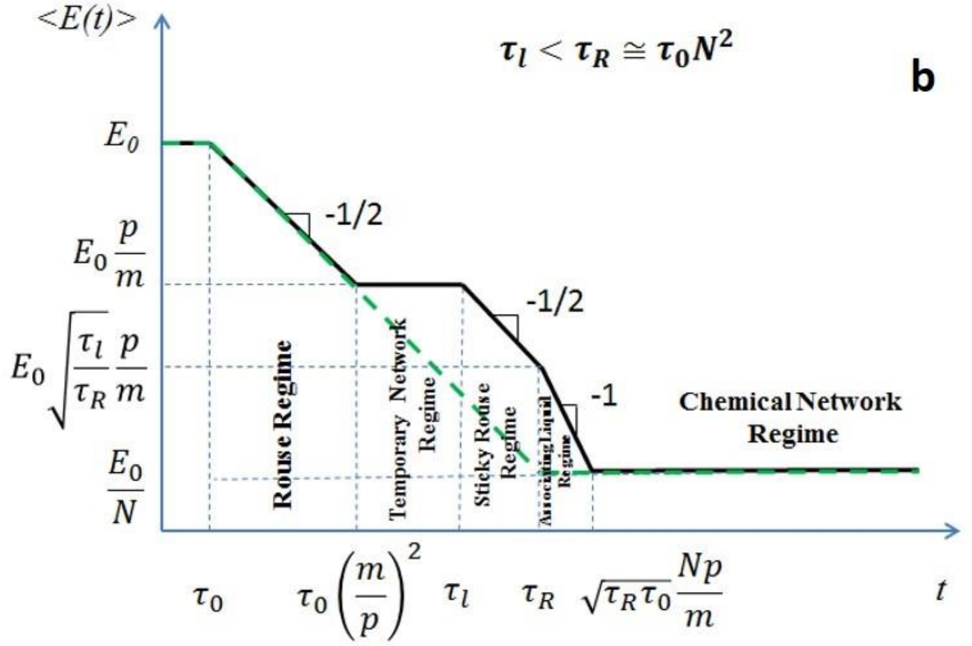

Supplementary Figure 2 | Relaxation of reversible network. Dependence of the time average Young's modulus $\langle E(t)\rangle = t^{-1}\int_0^t E(\tau)d\tau$ on time t for associating networks (black line) and chemical networks (green dashed line), for two temperature intervals for which (a) $\tau_l > \tau_R$ and (b) $\tau_0(m/p)^2 < \tau_l < \tau_R$, respectively. Logarithmic scales.

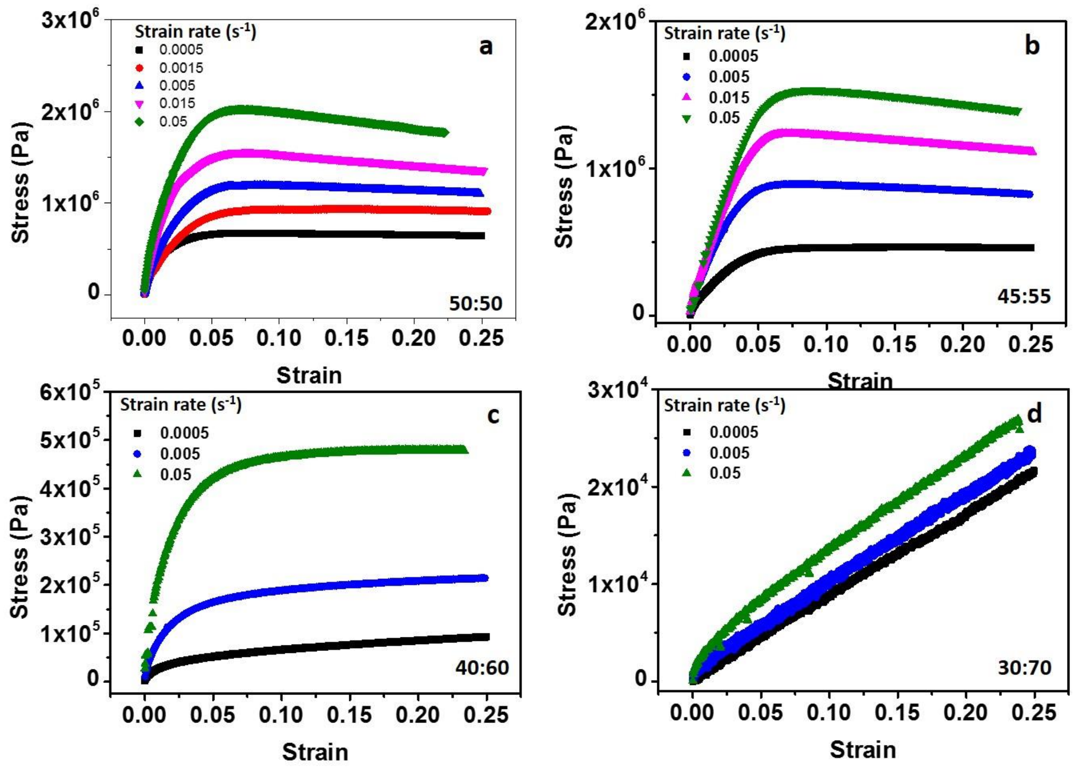

Supplementary Figure 3 | Different compositions and strain rates. Stress-strain curves for different MAAc:DMAA molar ratios (a) 50:50; (b) 45:55; (c) 40:60; (d) 30:70 samples were measured in silicone oil at 3°C with different strain rates. Lower MAAc concentration leads to lower density and strength of hydrogen bonds and thus lower stress.

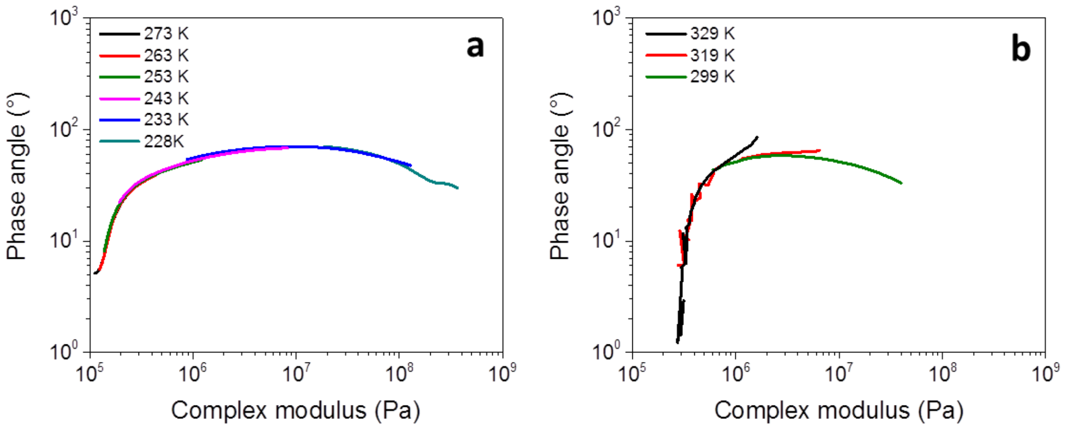

Supplementary Figure 4 | Failed time-temperature superposition. VGP plots of two polymeric materials measured at varying temperatures in a range of frequencies from 0.1 to 100 Hz. (**a**) Master curve of linear poly(n-butyl acrylate) displaying excellent overlap of data between temperatures in accordance with the time temperature superposition principle. (**b**) Master curve of 45:55 MAAc:DMAA gel displaying deviation between data at different temperatures indicating multiple temperature dependencies within relaxation processes.

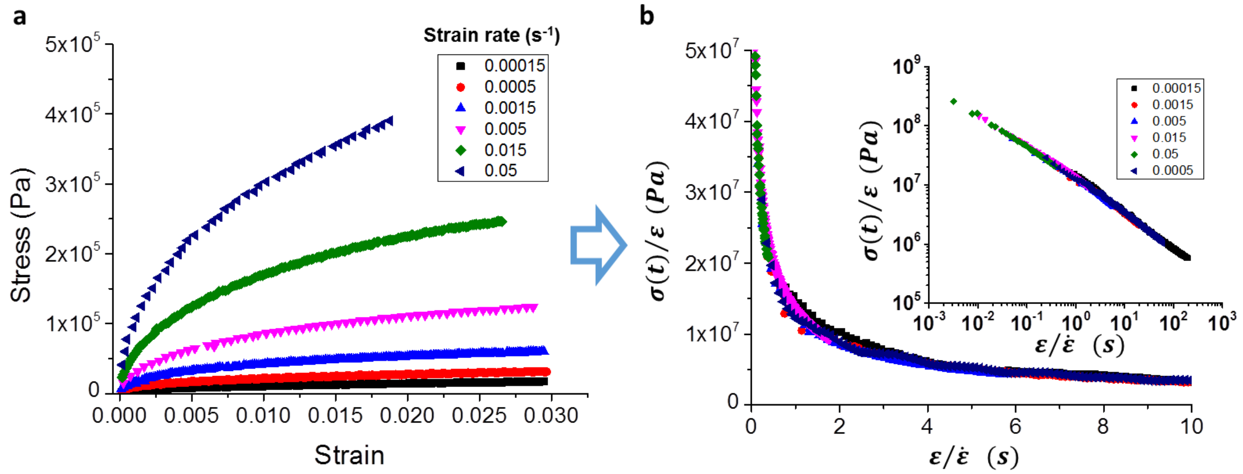

Supplementary Figure 5 | Linear viscoelasticity. The time-average network Young's modulus $(\langle E(t)\rangle = t^{-1}\int_0^t E(\tau)d\tau = \sigma(t)/\varepsilon)$ as a function of time $t = \varepsilon/\dot{\varepsilon}$ was extracted from (a) stress-strain curves measured at different strain rates at 25°C in silicone oil and (b) plotted according to Supplementary Equation 29. In small strain range, the perfect superposition of the $\langle E(t)\rangle$ curves in (b) validates the linear viscoelasticity approximation.

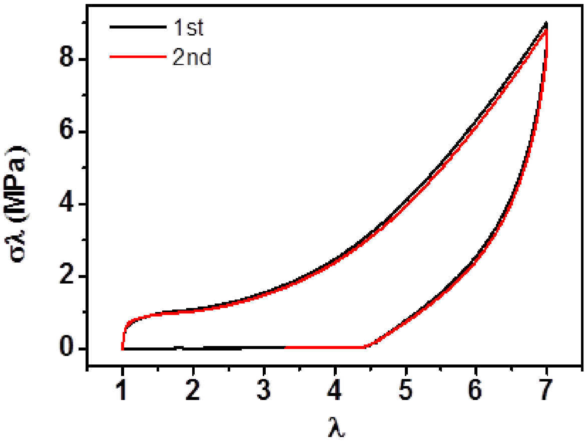

Supplementary Figure 6 | High extensibility and reversibility. A dogbone-shaped sample (MAAc:DMAA = 50:50) was stretched to a strain of 600% at a rate of 0.05 s^{-1} at 22 ^{o}C with DMA, then unloaded at the same rate. After 4 h in silicone oil, the sample fully restored its initial length and mechanical properties as shown by the second loading-unloading cycle conducted under the same conditions.

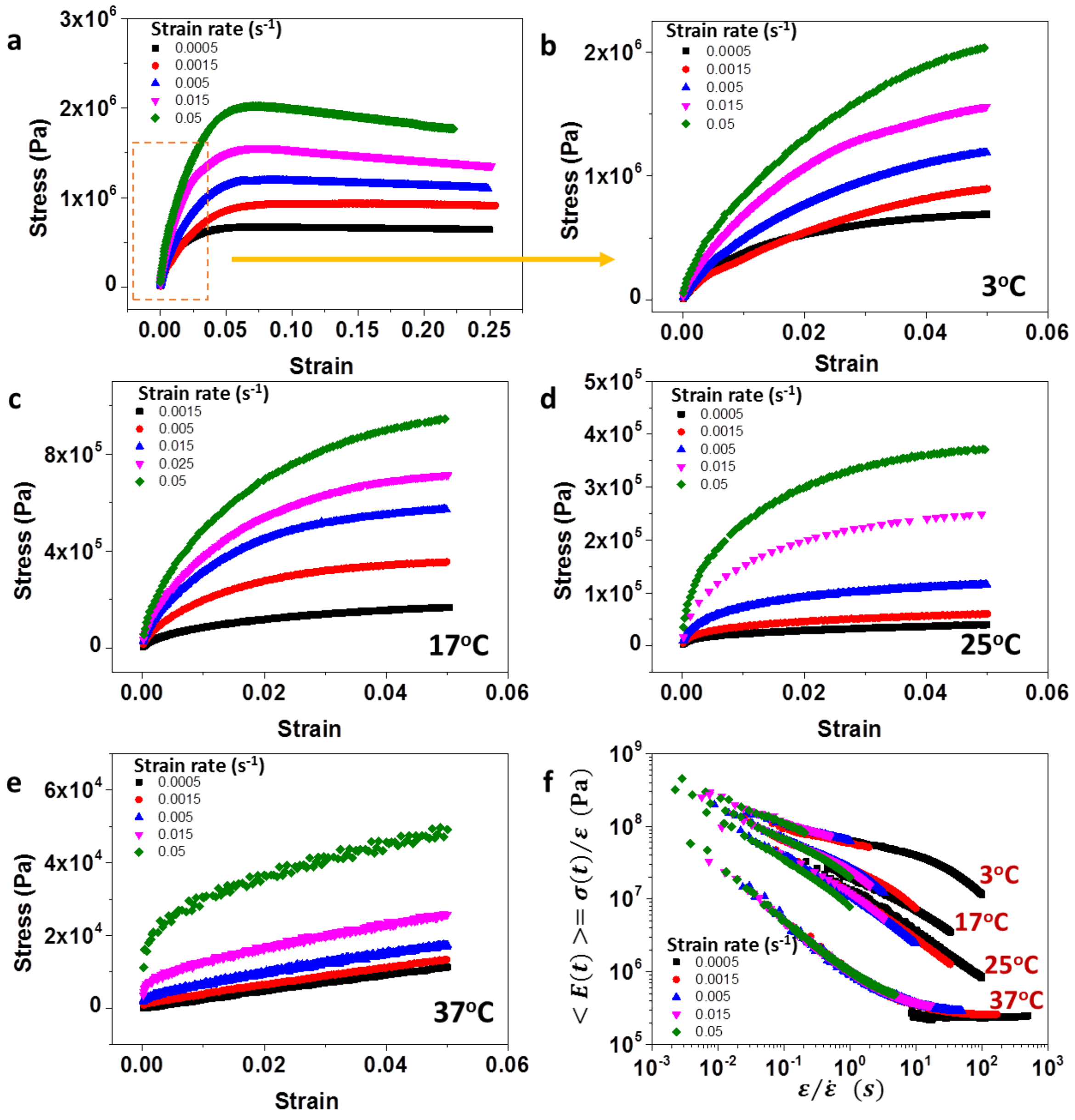

Supplementary Figure 7 | Stress-strain at different temperatures and strain rates. The stress-strain curves of dogbone-shaped samples (MAAc:DMAA = 50:50) were measured in silicone oil at different temperatures: (**a-b**) 3°C; (**c**) 17 °C; (**d**) 25 °C; (**e**) 37 °C and different strain rates from 0.0005 s^{-1} to 0.05 s^{-1}. (**f**) Time-average network Young's modulus ($\langle E(t)\rangle = t^{-1}\int_0^t E(\tau)d\tau = \sigma(t)/\varepsilon$) collapses different strain rates in small strain (~0.05) within the limits of linear approximation. The modulus decreases with temperature due to dissociation of hydrogen-bonded crosslinks.

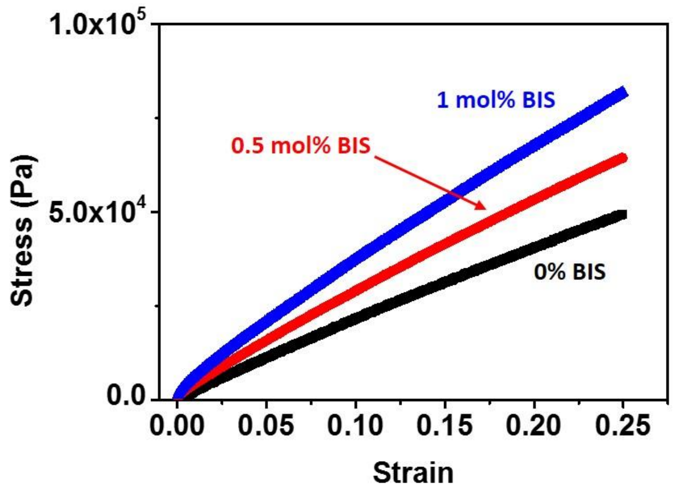

Supplementary Figure 8 | Effect of chemical cross-linking on modulus. Stress-strain curves of samples (MAAc:DMAA = 50:50) with different concentrations of cross-linker, *N,N'*-methylenebisacrylamide (BIS). Dogbone-shaped samples were stretched at 37 ^{o}C with a strain rate of 0.0005 s^{-1}. The high temperature and low strain rate almost eliminate the contribution of H-bonding to modulus.

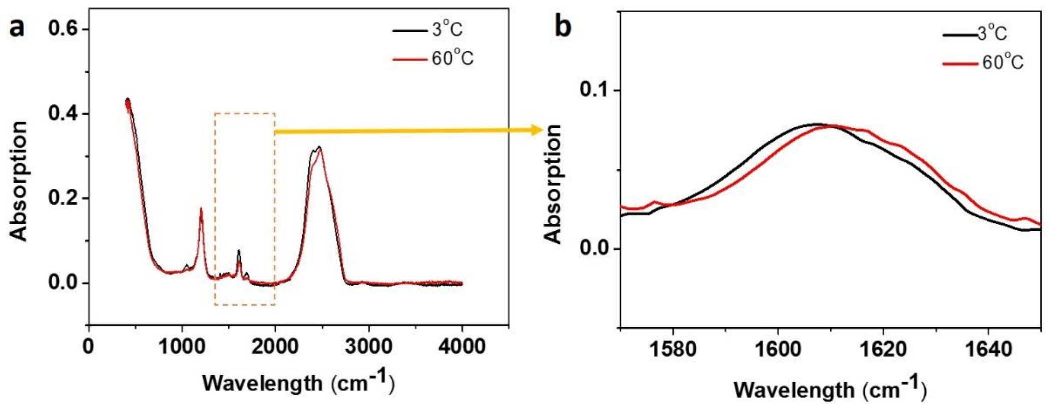

Supplementary Figure 9 | FTIR study on hydrogen bonds. (**a**) FTIR-ATR spectra of hydrogel at 3 ^{o}C and 60 ^{o}C. Samples (MAAc:DMAA = 50:50) were prepared in D_2O to eliminate absorption peak of H_2O. During measurements, the sample was immersed in D_2O solvent to maintain constant temperature and eliminate evaporation. (**b**) Zoomed-in bands corresponding to C=O group of DMAA. The high-frequency shift of the peak maximum at 60 ^{o}C suggests the dissociation of H-bonds at high temperature.

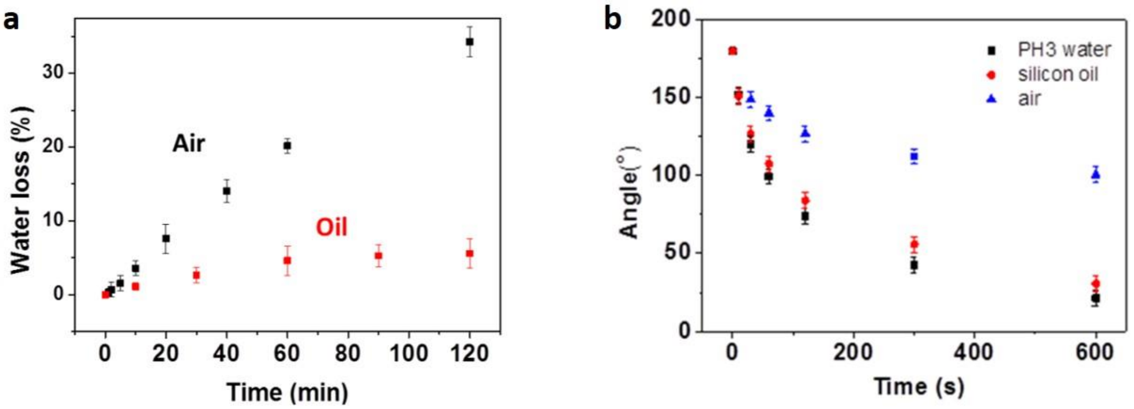

Supplementary Figure 10 | Effect of water evaporation on shape recovery. (a) Two pieces of hydrogels (MAAc:DMAA = 50:50, 15mm×5mm×1mm) were stored in open air or in silicone oil at room temperature (~22 ^{o}C). The water loss was measured as $\Delta m/0.7m_0 \times 100\%$, where Δm is weight change and m_0 is the initial weight of gel, 0.7 is the water fraction in the original sample. Compared to the measurement in open air, the water loss with oil protected sample was negligible (some evaporation occurs during weight measurement in air). The error bar indicates the standard deviation of the average of three separate experiments **(b)** Unfolding of a hairpin in different environments (air, pH3 water, and silicone oil) as indicated. The hairpin was programmed by folding a straight rod of the 50:50 MAAc:DMAA hydrogel in pH3 water for 1 min at 22 °C.

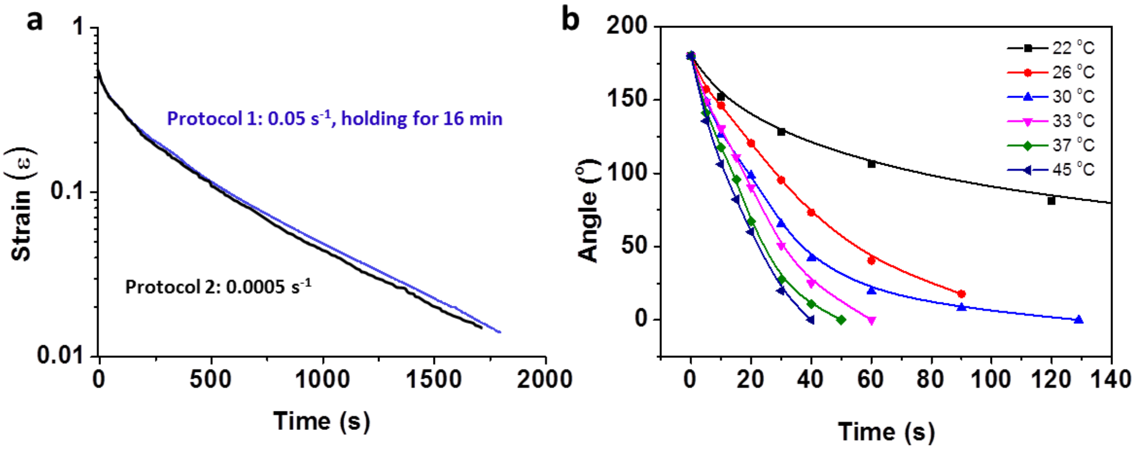

Supplementary Figure 11 | Effect of different programming protocols and temperature on the recovery rate. (a) Kinetics of shape recovery (50:50 MMAc-*co*-DMAA gel) for different programming protocols as indicated but with the same total programming time. They show similar shape recovery rate. **(b)** Hydrogel hairpin samples (MAAc:DMAA = 50:50) were bent 180° and programmed for 15 min in silicone oil at 22 ^{o}C and then recovered in oil at different temperatures. The angles were measured and plotted as a function of time for each recovery

temperature. The faster recovery at higher temperatures is ascribed to faster dissociation of hydrogen bonds.

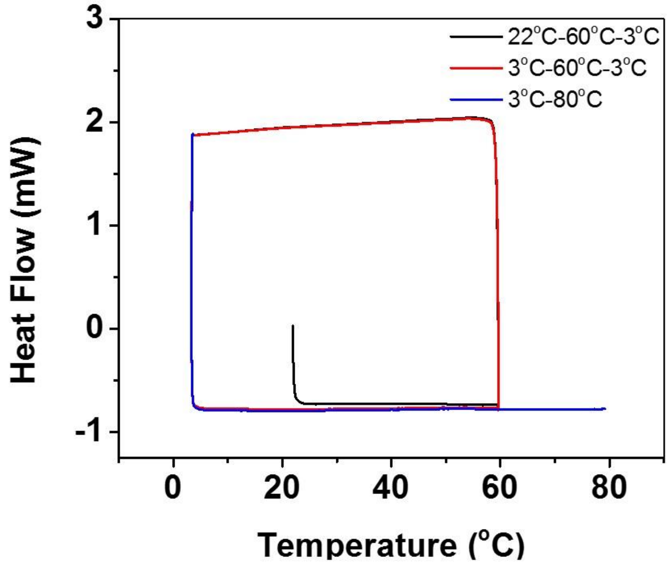

Supplementary Figure 12 | Lack of thermal transitions. DSC scanning for wet hydrogel (MAAc:DMAA = 50:50) performed at a heating and cooling rate of 2 °C/min for 3 cycles (22 °C -60 °C -3 °C, 3 °C -60 °C -3 °C, and then 3 °C to 80 °C).

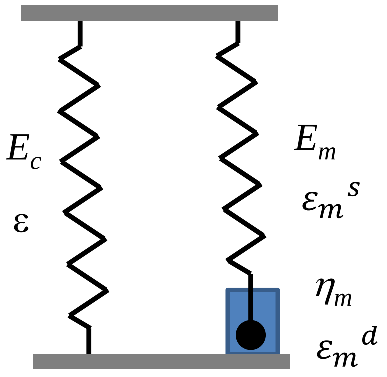

Supplementary Figure 13 | The standard linear solid model for description of relaxation in reversible network.

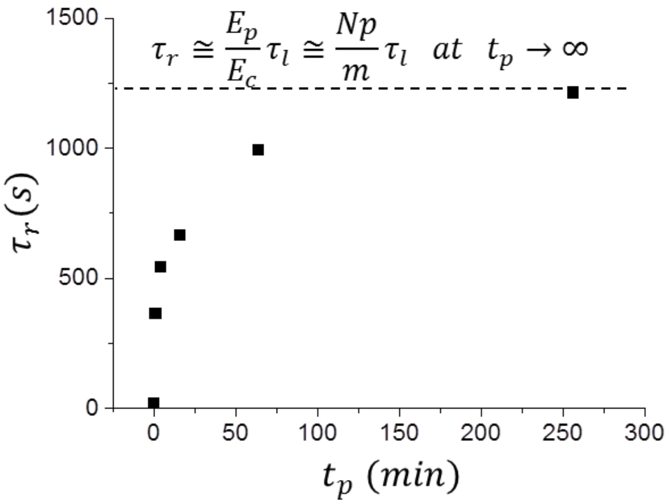

Supplementary Figure 14 | **Recovery rate vs programming time.** From Fig. 3a in the main text, we obtain the strain recovery time $\boldsymbol{\tau_r}$, which increases with the programming time $\boldsymbol{t_p}$ for a sample programmed by Protocol 2: uniaxial extension, $\dot{\varepsilon} = 0.05\ \mathrm{s}^{-1}$,$\varepsilon = 50\%$, $T = 25$ ℃.

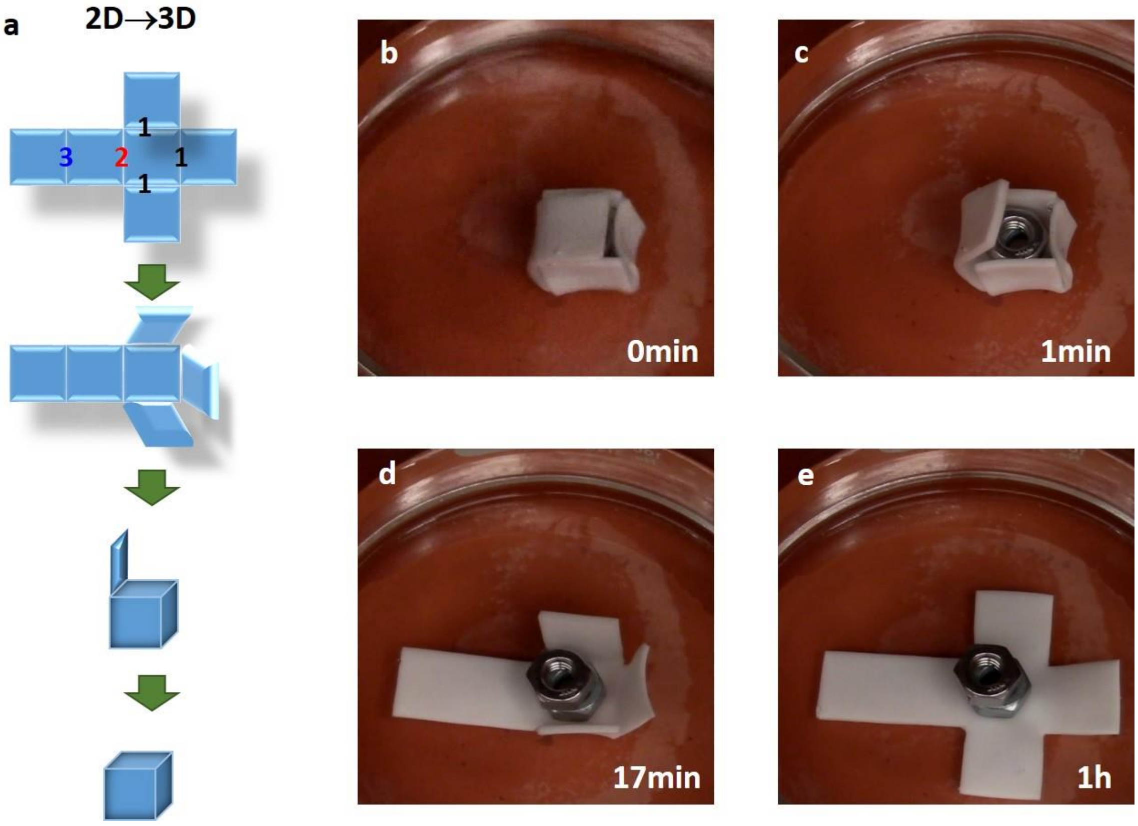

Supplementary Figure 15 | Sequential opening of a box. (a) Programming of cargo carrying box cut from a gel sheet (MAAc:DMAA = 50:50, 70 wt% water). The three short sides (1) were first folded to 90° in air, followed by the long side (2) and finally the top lid (3). each with different programming times: (3) 30 min, (2) 10 min, (1) 1 min, respectively. (b-e) Sequential opening of the box (pH 3 water, 22 °C): each side of the box unfolds in the reverse order of the programming time (longer programming time leads to slower recovery).

Supplementary Discussion

Theoretical Analysis

1. Thermodynamics of association

Since an equilibrium between separated groups A, B and their complex C is controlled by hydrogen bond association/dissociation, the reaction describing this process is

$$\mathrm{A} + \mathrm{B} \Leftrightarrow \mathrm{C} \qquad (1)$$

with an association reaction constant K_{ass} and a dissociation reaction constant K_{dis}. The evolution of concentration of the associated species [C] in a system is governed by the following kinetic equation

$$\frac{d[C]}{dt} = -K_{\mathrm{dis}}[C] + K_{\mathrm{ass}}[A][B] \qquad (2)$$

In an equilibrium state, the rate of change of [C] is equal to zero and the equilibrium concentration of complexes is given by

$$\frac{[C]}{[A][B]} = \frac{K_{\mathrm{ass}}}{K_{\mathrm{dis}}} = \nu \exp\left(-\frac{E_{\mathrm{ass}} - E_{\mathrm{dis}}}{kT}\right) \qquad (3)$$

where concentrations $[A]=[A_0]-[C]$ and $[B]=[B_0]-[C]$, ν is the association volume (on the order of the pervaded volume per associating pair), E_i are activation energies of forward and reverse reactions (Supplementary Figure 1), k is the Boltzmann constant and T is the absolute temperature. In a special case, when initial concentrations of A and B are the same, we can introduce a parameter, degree of conversion $p=[C]/[A_0]$, and simplify Supplementary Equation 3

$$\frac{p}{(1-p)^2} = [A_0]\nu \exp\left(\frac{\Delta\varepsilon}{kT}\right) \qquad (4)$$

where $\Delta\varepsilon = E_{\mathrm{dis}} - E_{\mathrm{ass}}$, energy difference between associated and dissociated states. The presence of the associating bonds results in effective attractive second virial coefficient between associating groups. In the presence of associating groups, the free energy of the polymer system responsible for the interactions between monomers has the following form[1]

$$\frac{F_{\mathrm{int}}}{kT} = \frac{V}{\nu}\left[\frac{\phi^2}{2} + \frac{\phi^3}{6} + \frac{\phi}{m}\left(\frac{p}{2} + \ln(1-p)\right)\right] \approx \frac{V}{\nu}\left[\left(1 - \frac{1}{m^2}\exp\left(\frac{\Delta\varepsilon}{kT}\right)\right)\frac{\phi^2}{2} + \frac{\phi^3}{6}, \qquad \textit{for } p<1 \qquad (5)$$

where m is the average number of monomers between associating groups and ϕ is polymer volume fraction in the solution. Thus during network polymerization process, as concentration of associating groups increases, the system will phase separate into polymer-rich and polymer-poor phases. This is manifested as a sample becoming milky.

The degree of polymerization of the polymeric strands between associating groups is controlled by the degree of conversion, p. For a polymeric strand to have n unassociated groups in a row starting with bonded pair, the probability is equal to

$$w(n) = (1-p)^n p \tag{6}$$

where factor (*1-p*) describes probability that associating group is unassociated and p is a probability for associating groups to form a bond. Note that the probability $w(n)$ is normalized such that

$$\sum_{n=0}^{\infty}(1-p)^n p = \frac{p}{1-(1-p)} = 1 \tag{7}$$

In substituting the upper limit for the sum by infinity, we have assumed that the number of associating groups per polymeric strand $n_{st}>>1$. The average number of monomers in a section of polymeric strand with n unassociated groups is equal to

$$\langle N\rangle = m\sum_{n=0}^{\infty}(n+1)(1-p)^n p = \frac{m}{p} \tag{8}$$

Probability $w(n)$ can be expressed in terms of concentration $c(n)$ of polymeric strands with n-open stickers

$$w(n) = c(n)/\sum_{n=0}^{\infty} c(n) \tag{9}$$

Total concentration of n-mers can be found by taking into account its relation with total monomer concentration ρ

$$\rho = \sum_{n=0}^{\infty}(n+1)mc(n) = m(\sum_{s=0}^{\infty} c(s))\sum_{n=0}^{\infty}(n+1)(1-p)^n p = (\sum_{s=0}^{\infty} c(s))m/p \tag{10}$$

We can use Supplementary Equation 10 to rewrite concentration of n-mers, $c(n)$ in terms of probability $w(n)$ and total monomer density

$$c(n) = \frac{\rho}{m}p^2(1-p)^n \tag{11}$$

Below we will use this distribution function to calculate stress evolution in polydisperse associating network.

2. Dynamics of associating networks

In polymeric networks, dynamics of strands forming a network on the time scales t smaller than the strands' Rouse time, $\tau_R = \tau_0 N^2$ (N - degree of polymerization (DP) of network strands and τ_0 - characteristic monomer time), is that of a melt of polymer chains with the same DP. At these time scales, the dynamics of polymer chains is not influenced by crosslinks and network modulus decays with time as, $E(t) \propto \rho kT(\frac{\tau_0}{t})^{1/2}$, where ρ is a monomer number density. Here we consider $N<N_e$ (entanglement DP). However, at the time scales t larger than the Rouse time of polymeric strands the network response is pure elastic with Young's modulus $E \propto \rho kT/N$. We will use the following approximation for time dependent Young's modulus to describe network relaxation:

$$E(t) \approx 3\rho kT\begin{cases}(\tau_0/t)^{1/2}, & for \quad \tau_0 < t \le \tau_R = \tau_0 N^2 \\ 1/N, & for \quad \tau_R \le t\end{cases} \tag{12}$$

Using Boltzmann superposition principle,[2] we can describe stress evolution in polymeric networks undergoing uniaxial elongation at a constant strain rate $\dot{\varepsilon}$ as

$$\sigma(t) = \int_0^t E(t-t')d\varepsilon(t') = \int_0^t E(t-t')dt' = \dot{\varepsilon}\int_0^t E(\Delta t)d\Delta t \qquad (13)$$

For the stress relaxation modulus given by Supplementary Equation 12, integration of Supplementary Equation S13 results in

$$\sigma(t) \approx 2E_0\dot{\varepsilon}\tau_0^{1/2}t^{1/2}, \qquad for\ t < \tau_\mathrm{R} \qquad (14)$$

where we introduced $E_0 = 3kT/v$. For experiments at constant strain rate, we substitute $t = \varepsilon/\dot{\varepsilon}$ into Supplementary Equation 14 to express $\sigma(t)$ as a function of strain ε as

$$\sigma(\varepsilon) \approx 2E_0\tau_0^{1/2}\dot{\varepsilon}^{1/2}\varepsilon^{1/2}, \qquad for\ t < \tau_\mathrm{R} \qquad (15)$$

In the opposite limit, $t > \tau_\mathrm{R}$,

$$\sigma(\varepsilon) \approx 2E_0\dot{\varepsilon}\tau_\mathrm{R}/N + E_0\dot{\varepsilon}(t-\tau_\mathrm{R})/N \approx E_0\varepsilon/N\,, for\ t \geq \tau_\mathrm{R} \qquad (16)$$

Polydisperse networks have a distribution of the Rouse times such that crossover from the unentangled melt to network relaxation regime depends on chain's DP. For associating networks, it depends on DP of polymeric strands between associating bonds. Note that in addition to Rouse time of such strands there is also another time scale τ_l (life-time of the bond), which determines the characteristic time scale when associating network begins to change bond arrangements. Here we assume that the dissociation process of physical crosslinks is a limiting step of the association/dissociation reaction (see Supplementary Figure. 1) such that $\tau_\mathrm{l} = \tau_a \exp(E_a/kT)$, where E_a=E_dis. On the time scales $t < \tau_\mathrm{l}$, each associating bond is considered to be permanent bond. In this case, the concentration of strands with n-open associating groups is given by Supplementary Equation 11. Here we assume that there is time scale separation $\tau_\mathrm{l} > \tau_\mathrm{R} = \tau_0 N^2$ (Rouse time of the polymeric strands of the chemical network). In a polydisperse sample at time t, chains can be divided into two groups: (i) the chains with DP = N, such that their Rouse time $\tau_0 N^2 > t$, these chains contribution to the system modulus is time dependent; (ii) chains with $\tau_0 N^2 \leq t$, which at these time scales provide a pure elastic contribution to network modulus. In the Rouse regime, network relaxation dynamics is controlled by sections of the chains containing l monomers for which $t \approx \tau_\mathrm{k} \approx \tau_0 l^2 \Rightarrow l \approx (t/\tau_0)^{1/2}$. In average, each such section stores energy on the order of thermal energy kT. Therefore, contribution of n-mers with concentration $c(n)$ to system modulus will be $c(n)m(n+1)/l \approx c(n)m(n+1)(\tau_0/t)^{1/2}$. Combining contributions from all n-mers we have

$$\sigma(t) = 3\dot{\varepsilon}kT\sum_{n=\sqrt{t/\tau_m}-1}^{\infty} c(n)m(n+1)\int_0^t d\Delta t(\tau_0/\Delta t)^{1/2} + 3\dot{\varepsilon}kT\sum_{n=0}^{\sqrt{t/\tau_m}-1} c(n)m(n+1)\int_0^{\tau(n)} d\Delta t(\tau_0/\Delta t)^{1/2} + 3\dot{\varepsilon}kT\sum_{n=0}^{\sqrt{t/\tau_m}-1} c(n)\int_{\tau(n)}^t d\Delta t, for\ t < \tau_\mathrm{l} \qquad (17)$$

where we introduced $\tau_{\mathrm{m}}=\tau_0 m^2$ and $\tau(n)=\tau_{\mathrm{m}}(n+1)^2$. After substitution of Supplementary Equation 11 for $c(n)$, Supplementary Equation 17 transforms into

$$\sigma(t)=2E_0\dot{\varepsilon}\sqrt{\tau_0 t}\sum_{n=\sqrt{t/\tau_{\mathrm{m}}}-1}^{\infty}(n+1)p^2(1-p)^n+2E_0\dot{\varepsilon}\sqrt{\tau_0\tau_m}\sum_{n=0}^{\sqrt{t/\tau_{\mathrm{m}}}-1}(n+1)^2p^2(1-p)^n$$

$$+E_0\dot{\varepsilon}m^{-1}\sum_{n=0}^{\sqrt{t/\tau_{\mathrm{m}}}-1}p^2(1-p)^n\left(t-\tau_m(n+1)^2\right), for\ t<\tau_{\mathrm{l}} \qquad (18)$$

In the case when conversion $p < 1$, we can substitute summation in Supplementary Equation 18 by integration

$$\sigma(t)\approx 2E_0\dot{\varepsilon}\sqrt{\tau_0 t}\int_{\sqrt{t/\tau_{\mathrm{m}}}}^{\infty}dn n\langle n\rangle^{-2}\exp(-n/\langle n\rangle)+2E_0\dot{\varepsilon}\sqrt{\tau_0\tau_{\mathrm{m}}}\int_0^{\sqrt{t/\tau_{\mathrm{m}}}}dn n^2\langle n\rangle^{-2}\exp(-n/\langle n\rangle)$$

$$+E_0\dot{\varepsilon}m^{-1}\int_0^{\sqrt{t/\tau_{\mathrm{m}}}}dn\langle n\rangle^{-2}\exp(-n/\langle n\rangle)(t-\tau_{\mathrm{m}}n^2),\ for\ \ t<\tau_{\mathrm{l}} \qquad (19)$$

where $< n >= 1/p$. Performing integration in Supplementary Equation 19, we obtain

$$\sigma(t)\approx\frac{E_0 p}{m}\dot{\varepsilon}\tilde{\tau}_R\left(t/\tilde{\tau}_{\mathrm{R}}+2\left(1-\exp\left(-\sqrt{t/\tilde{\tau}_{\mathrm{R}}}\right)\right)\right), for\ \ t<\tau_{\mathrm{l}} \qquad (20)$$

Thus the stress evolution in a polydisperse network depends on the Rouse relaxation time of an average strand between associated bonds with relaxation time $\tilde{\tau}_{\mathrm{R}}=\tau_0 m^2/p^2$.

On the time scales $t\geq\tau_{\mathrm{l}}$, associating bonds cannot be considered as permanent crosslinks and $c(n)$ of n-mers begins to change. To describe this regime, we use Tobolsky's dual network idea[3], assuming that network modulus changes with the degree of conversation p. This also means that relaxation of a combined strand after dissociation takes place is much faster than the occurrence of the next bond dissociation event (time scale separation assumption, $\tau_{\mathrm{l}}>\tau_{\mathrm{R}}$). Survival probability of an associated bond over time interval Δt is $(1-\Delta t/\tau_{\mathrm{l}})$. For a bond to survive over time interval t, the probability is estimated as $\mathrm{T}(t)=\prod_i(1-\Delta t_{\mathrm{i}}/\tau_{\mathrm{l}})\approx\exp(-\sum_i\frac{\Delta t_{\mathrm{i}}}{\tau_{\mathrm{l}}})\approx\exp(-t/\tau_{\mathrm{l}})$. $T(t)$ gives a transition probability, therefore evolution of the conversion over time is also an exponential function of time $p(t)\approx pexp(-t/\tau_{\mathrm{l}})$. Taking this into account, we can rewrite Supplementary Equation 17 as follows

$$\sigma(t)=\sigma(t_{\mathrm{c}})+3\dot{\varepsilon}kT\sum_0^{\infty}\int_{t_{\mathrm{c}}}^{t}c(n,t)d\Delta t\approx\sigma(t_c)+3\dot{\varepsilon}kT\frac{\rho}{m}\int_{t_{\mathrm{c}}}^{t}\sum_{n=0}^{\infty}p(\Delta t)^2\left(1-p(\Delta t)\right)^n d\Delta t$$

$$\approx\sigma(t_{\mathrm{c}})+E_0 m^{-1}\int_{t_{\mathrm{c}}}^{t}pexp(-\Delta t/\tau_{\mathrm{l}})d\Delta t$$

$$\approx \sigma(t_c) + \frac{E_0 \dot{\varepsilon} p \tau_l}{m} exp(-t_c/\tau_l)\left(1 - \exp\left(-\frac{t - t_c}{\tau_l}\right)\right), for\ t \geq t_c \tag{21}$$

where t_c is a reference time, $\tilde{\tau}_R < t_c < \tau_l$, which provides continuity of the stress given by Supplementary Equations 20 and 21. On average, each polymeric strand of the degree of polymerization *N* between crosslinks has *Np/m* associated reversible bonds at time $\approx \tau_l$. For all these bonds to dissociate would require time on the order of $\tau_l Np/m$. At these time scales the network modulus approaches that of a chemical network consisting of strands with degree of polymerization between crosslinks *N*.

$$\frac{\sigma(t)}{\varepsilon} \approx E_0 \frac{\tau_l p}{m} \frac{\dot{\varepsilon}}{\varepsilon} \approx \frac{E_0}{N} \Longrightarrow t \approx \tau_l \frac{Np}{m} \tag{22}$$

In Supplementary Figure 2, we summarize our results for the time average Young's modulus

$$\langle E(t) \rangle = \frac{\sigma(t)}{\varepsilon} = \frac{\dot{\varepsilon}}{\varepsilon} \int_0^t E(\tau) d\tau = \frac{1}{t} \int_0^t E(\tau) d\tau \tag{23}$$

in different network deformation regimes. At time scales $\tau_0 \leq t \leq \tilde{\tau}_R = \tau_0 (m/p)^2$, the Rouse modes of polymeric strands between associating bonds determine time dependence of the time average network Young's modulus ("Rouse" Regime).

$$\langle E(t) \rangle = E_0 (\tau_0/t)^{1/2} , \quad for \quad \tau_0 \leq t \leq \tau_0 (m/p)^2 \tag{24}$$

In this time interval, behavior of reversible network is similar to that of a chemical network. Crossover to "Temporary Network" regime takes place at $t \approx \tau_0 (m/p)^2$. In this regime associated bonds could be considered as permanent crosslinks since their life time τ_l is longer than an experimental time scale *t*. The network modulus in this regime is

$$\langle E(t) \rangle \approx E_0\, p/m , \quad for \quad \tau_0 (m/p)^2 \leq t \leq \tau_l \tag{25}$$

Temporary network of associating bonds start to evolve at $t \approx \tau_l$. In the time interval $t > \tau_l$, the network modulus is determined by breaking of the associated bonds, which effectively results in increase of the degree of polymerization of the temporary network strands ("Bond Breaking" regime). The network modulus in this regime is inversely proportional to time

$$\langle E(t) \rangle \approx E_0 \frac{\tau_l p}{m} t^{-1}, \quad for \ \tau_l \leq t \leq \tau_l N\, p/m \tag{26}$$

Finally, all unrelaxed associated bonds disappear at time scales on the order of $\tau_l N\, p/m$ and network elastic response is that of a chemical network ("Chemical Network" regime) with modulus

$$\langle E(t) \rangle \approx E_0/N, \quad for\ \tau_l N\, p/m \leq t \tag{27}$$

Increasing temperature will result in decrease of the degree of conversion p (see Supplementary Equation 4) and decrease of the life time τ_l of the associated bonds, $\tau_l = \tau_a \exp(E_a/kT)$. This is manifested in Supplementary Figure 2 as shift of the location of the crossover to the temporary plateau regime with value of the network modulus $E_0 p/m$ down and to the right along the dashed line describing time dependence of modulus of chemical networks of the same degree of

polymerization between crosslinks. At the same time, the location of the "Associating Liquid" regime with $\langle E(t)\rangle \propto t^{-1}$ will shift to the left since the life time of the associating bond will decrease exponentially with temperature. A new time dependent regime will appear when the life time of the bond τ_l becomes shorter than the Rouse time of the polymeric strand between crosslinks τ_R (see Supplementary Figure. 2b). For such temperature range, after bond dissociation the relaxation of the enlarged strands between associated bonds will follow a Rouse-like relaxation with a characteristic time τ_l ("Sticky Rouse" regime). Therefore, at time interval $\tau_l \leq t \leq \tau_R$ the time dependent network modulus has the following form

$$\langle E(t)\rangle \approx E_0\, p/m \left(\frac{\tau_l}{t}\right)^{1/2}, \qquad for\ \tau_l \leq t \leq \tau_R \tag{28}$$

In order to demonstrate how the time average Young's modulus depends on strain and strain rate, it is convenient to rewrite Supplementary Equation 15 as

$$\langle E(\varepsilon)\rangle = \frac{\sigma(\varepsilon)}{\varepsilon} \cong E_0{\tau_0}^{1/2}\left(\frac{\varepsilon}{\dot{\varepsilon}}\right)^{-1/2} \tag{29}$$

This expression suggests that all stress-strain curves shown in Supplementary Figure. 5a can be collapsed on one universal curve by plotting σ/ε vs $\varepsilon/\dot{\varepsilon}$ (Supplementary Figure 5b), which is consistent with the predictions of the Rouse model (Supplementary Equation 24).

3. Two state model of associating network relaxation

Analysis of the associating networks dynamics shows that in a wide time range, we can approximate network properties by a two network model, with one being formed by physical crosslinks with a life time τ_l and plateau modulus E_p, and another network being network of permanent chemical crosslinks with modulus E_c. In this approximation, we may neglect Rouse-like relaxation of polymeric strands between physical crosslinks which occur at short time scales. Phenomenologically, dynamics of such system can be described by "standard linear solid" model (Supplementary Figure. 13) of elastic spring with modulus E_c connected in parallel with a Maxwell element having $E_m = E_p - E_c$ and $\eta_m = E_m\tau_l$. Since for our networks the ratio of the Young's modulus of the physical network and that of a chemical network is about 10^3, the small deformation of the physical network strands could result in large deformations of the strands of chemical network. For this reason, to account for this large deformation of the chemical network, we will use a general stress-strain relation

$$\sigma_c(t) = \frac{E_c}{3}\left(\lambda(t)^2 - \lambda(t)^{-1}\right) \tag{30}$$

where $\lambda(t) = 1 + \varepsilon(t)$ - deformation ratio.

To analyze dynamics of the shape recovery process, we consider a strain relaxation from a state with initial strain ε_0. Since the external stress is zero, we can write

$$\frac{E_c}{3}\left(\lambda(t)^2 - \lambda(t)^{-1}\right) = -E_m\varepsilon_m^s(t) \tag{31}$$

Differentiating both sides of the Supplementary Equation 31 with respect to time, we obtain the rate of deformation of the Maxwell spring

$$\dot{\varepsilon}_m^s(t) \approx -\frac{E_c}{E_m}\dot{\varepsilon}(t) \tag{32}$$

Partitioning of deformation in the Maxwell element between spring and dashpot (see Supplementary Figure 13) allows us to write the following expression for the whole sample rate of deformation

$$\dot{\varepsilon}(t) = \dot{\varepsilon}_m^s(t) + \dot{\varepsilon}_m^d(t) \approx -\frac{E_c}{E_m}\dot{\varepsilon}(t) + \dot{\varepsilon}_m^d(t) \tag{33}$$

Taking into account a Maxwell element

$$\eta_m\dot{\varepsilon}_m^d(t) = E_m\varepsilon_m^s(t) = -\frac{E_c}{3}(\lambda(t)^2 - \lambda(t)^{-1}) \tag{34}$$

we obtain

$$\dot{\varepsilon}(t) = -\frac{E_c E_m}{3(E_c+E_m)\eta_m}(\lambda(t)^2 - \lambda(t)^{-1}) \tag{35}$$

Internal stress in a sample is manifested in exponential shift of the life time of the physical bond. In the framework of the Eyring's assumption of the relaxation time shift due to internal stress, one can write deformation dependent life time

$$\tau(\lambda(t)) \approx \tau_l exp\left(-\frac{vE_c\left(\lambda(t)^2-\lambda(t)^{-1}\right)}{3kT}\right) \tag{36}$$

where v is the activation volume. Substitution of this equation into Supplementary Equation 35 results in

$$\dot{\varepsilon}(t) = -\frac{\left(\lambda(t)^2-\lambda(t)^{-1}\right)}{3\tau_r} exp\left(\beta(\lambda(t)^2 - \lambda(t)^{-1})\right) \tag{37}$$

Here we introduce the effective relaxation time for strain recovery $\tau_r(t_p)$ and dimensionless parameter $\beta = vE_c/(3kT)$, where t_p – programming time, $E_c{\sim}N^{-1}$ and $E_p{\sim}\,p/m$ are the plateau moduli of the chemical and physical networks, respectively.

Analysis of the experimental data for strain recovery rate (Fig.3a) indicates that the characteristic recovery time τ_r increases with programing time t_p and then levels off as shown in Supplementary Figure 14. The shape recovery is driven by the strained chemical network (E_c) and resisted by a fraction $\phi(t_p)$ of re-associated physical cross-links, which increase with t_p. Note that in general case $\phi(t_p)$ is also a function of the programming sample deformation ε_p. Therefore, the recovery time in a standard linear solid model can be written as $\tau_r(t_p) =$

$\frac{\phi(t_{\mathrm{p}})E_{\mathrm{p}}}{E_{\mathrm{c}}}\tau_{\mathrm{l}}$, which accounts for rearrangement of the physical cross-links during the programming stage. In the limit of large t_{p}, the recovery time $\tau_{\mathrm{r}} = \frac{E_{\mathrm{p}}}{E_{\mathrm{c}}}\tau_{\mathrm{l}} \cong \frac{Np}{m}\tau_{\mathrm{l}}$ corresponds to the time $\frac{Np}{m}\tau_{\mathrm{l}}$ required for complete re-association of the physical network during its deformation (Supplementary Figure 7f).

We can apply this two state model to study stress relaxation in reversible network. In this case a constant strain ε_0 is applied to the sample. Using relation for the rate of change of strain in the Maxwell element

$$\dot{\varepsilon}_0 = 0 = \dot{\varepsilon}_{\mathrm{d}}(t) + \dot{\varepsilon}_{\mathrm{s}}(t) \rightarrow \dot{\varepsilon}_{\mathrm{d}}(t) = -\dot{\varepsilon}_{\mathrm{s}}(t) \tag{38}$$

we can rewrite the force balance condition for spring and dashpot as follows

$$\dot{\sigma}_{\mathrm{m}}(t) = -\frac{1}{\tau_{\mathrm{l}}}\sigma_m(t)exp\left(\frac{3\beta\sigma_{\mathrm{m}}(t)}{E_{\mathrm{c}}}\right) \tag{39}$$

In Supplementary Equation 39, exponential term accounts for the stress effect on the associating bond life time. Integration of Supplementary Equation 39 gives

$$E_1\left(\frac{3\beta\sigma_{\mathrm{m}}(0)}{E_{\mathrm{c}}}\right) - E_1\left(\frac{3\beta\sigma_{\mathrm{m}}(t)}{E_{\mathrm{c}}}\right) = \frac{t}{\tau_{\mathrm{l}}} \tag{40}$$

where function $E_1(x) = \int_x^\infty \frac{e^{-t}}{t}dt$. Note that instead of solving Supplementary Equation 40 for $\sigma_{\mathrm{m}}(t)$ it is more convenient to look at t as a function of stress. Time dependence of the stress in the Maxwell element is $\sigma_{\mathrm{m}}(t) = \sigma(t) - \sigma_\infty$.

Supplementary Methods

Materials

N,*N*-dimethylacrylamide (DMAA), methacrylic acid (MAAc), ammonium persulfate (APS), and *N,N,N'N'*-tetramethylethylenediamine (TEMED) were used as received (Sigma-Aldrich) without further purification. Water was produced by distillation and deionization to a resistance of 18 MΩ cm, followed by filtration through a 0.2 µm filter to remove particulate matter.

Hydrogel preparation

All hydrogels were prepared by a one-step copolymerization of DMAA and MAAc with different molar ratios, while the total monomer concentration is 33 wt%. A mixed aqueous solution of DMAA and MAAc was degassed with N_2 for 30 min. Then initiator APS and accelerator TEMED were added separately to the solution and the solution was then transferred to a glass mold with a PDMS spacer for polymerization at room temperature under N_2 atmosphere for 48h.

The dimethylamide group in DMAA is known to be a strong hydrogen-bond acceptor, while methacrylic acid is a potent hydrogen-bond donor. The copolymerization of DMAA and MAAc

leads to multiple intermolecular hydrogen bonding, which causes formation of polymer-rich aggregates stabilized by the hydrophobic interactions due to the presence of the α-methyl groups of PMAA. Besides, the polymerization process produces a low fraction of chemical cross-links due to the chain transfer reaction in copolymers with DMAA.[4] Thus, it yields a dual network hydrogel comprised of a dense H-bonded network integrated with a loose chemical network.

Dynamic Mechanical Analysis

The mechanical test was carried out on a Dynamic Mechanical Analysis (RSA-G2, TA Instrument) with an Immersion Clamps. Samples with a thickness of 1.6 mm were cut into dogbone shape (DIN 53504-S3, 2 mm in width with an initial length of 12 mm). In order to avoid the water evaporation during test, all of the mechanical tests were performed in silicone oil. Samples were stretched at a certain strain rate at a defined temperature. For shape programming, sample was stretched to a strain of 50% at a certain strain rate and then holding the sample at 50% strain for a defined time. Next, the sample was set to isoforce mode where the external force was set to be constantly at zero and then record the strain change over the recovery process.

Supplementary References

1. Semenov, A. N. & Rubinstein, M. Thermoreversible gelation in solutions of associative polymers. 1. Statics. *Macromolecules* **31**, 1373-1385 (1998).
2. Ward, I. M. & Sweeney, J. *Mechanical Properties of Solid Polymers*. (Wiley-Interscience, 1971).
3. Green, M. S. & Tobolsky, A. V. A New approach to the theory of relaxing polymeric media. *J. Chem. Phys.* **14**, 80-92 (1946).
4. Needles, H. L. & Whitfiel.Re. Crosslinking of Copolymers Containing N,N-Dimethylacrylamide. *J Polym Sci Part A* **3**, 3543-3548 (1965).
